# Cysteine-Rich Protein 61 (CCN1) Deficiency Alleviated Cardiac Remodeling in 5/6 Nephrectomized Mice by Suppressing the MAPK Signaling Pathway

**DOI:** 10.1155/cdr/6813183

**Published:** 2025-02-27

**Authors:** Yihan Zhao, Liang Gu, Yunxuan Chen, Yibei Lin, Jincheng Xing, Diyan Xu, Zhen Su, Zhouqing Huang

**Affiliations:** ^1^The Key Laboratory of Cardiovascular Disease of Wenzhou, Department of Cardiology, The First Affiliated Hospital of Wenzhou Medical University, Wenzhou, Zhejiang, China; ^2^Department of Cardiology, Sichuan Academy of Medical Sciences & Sichuan Provincial People's Hospital, School of Medicine, University of Electronic Science and Technology of China, Chengdu, Sichuan, China; ^3^Department of Nephrology, The First Affiliated Hospital of Wenzhou Medical University, Wenzhou, Zhejiang, China; ^4^Department of General Medicine, Mianyang Central Hospital, Mianyang, Sichuan, China

**Keywords:** 5/6 nephrectomy mice, cardiac remodeling, CCN1, MAPK signaling pathway

## Abstract

**Background:** With the progression of chronic kidney disease (CKD), we can often observe cardiac remodeling, fibrosis, and cardiac failure in patients. Cysteine-rich protein 61 (CCN1) is an extracellular matrix protein that plays a reuse role in cardiac remodeling. However, whether CCN1 participates in the crosslink between the heart and kidney in CKD and the potential mechanism remains unknown.

**Methods:** We constructed a mouse model of CKD by 5/6 nephrectomy (5/6 Nx). Hematoxylin–eosin staining (H&E), Masson's trichrome staining, and Sirius red staining were used to observe cardiac morphology and fibrosis. H9c2 cells were treated with si-CCN1 or si-NC or mitogen-activated protein kinase (MAPK)–related inhibitors or agonist before being cultured with 5/6 Nx mouse serum. The relative protein level was detected by Western blotting.

**Results:** We observed that CCN1 expression was markedly enhanced in the serum and heart tissues, accompanied by disordered myocardial arrangement, obvious cardiac fibrosis, hypertrophy, and decreased cardiac systolic function reflected by echocardiography. The relative markers collagen 1 (COL-1), transforming growth factor-*β* (TGF-*β*), heavy-chain cardiac myosin (MyHC), and atrial natriuretic peptide (ANP) presented an increase in expression. In vivo and in vitro, after the knockdown of CCN1, the above results in the CKD group or CKD serum group were reversed; in addition, the MAPK signaling pathway was obviously activated due to 5/6 Nx, which was abolished by CCN1 inhibition. CCN1 silencing or MAPK pathway inhibition also decreased the expression of myocardial fibrosis and hypertrophy markers in H9c2 cells, while MAPK-related agonist partly reversed the effect of CCN1 inhibition.

**Conclusion:** Our in vivo and in vitro study showed that specific CCN1 deficiency markedly alleviated cardiac remodeling in 5/6 Nx mice through the inhibition of the MAPK pathway.

## 1. Introduction

Chronic kidney disease (CKD) is a global public health problem, accounting for 10%–15% of the global adult population [1]. The prevalence of central vascular disease in patients with CKD is close to 70%. Cardiovascular disease is responsible for more than 50% of kidney disease–related deaths [2, 3]. Chronic primary kidney disease causes cardiac dysfunction, left ventricular hypertrophy, and increased adverse cardiovascular events, indicating a relationship defined as cardiorenal syndrome (Type IV) [4]. Mechanistically, CKD results in high cardiovascular complications due to the cumulative effects of hemodynamic overload, anemia, metabolic abnormalities, neuroendocrine dysregulation, and inflammatory activation [5–7].

CCN1 is a multifunctional extracellular matrix protein that is involved in cell adhesion, differentiation, senescence, apoptosis, and fibrosis [8–10]. It is constitutively expressed in mouse hearts, and systemic knockout of the CCN1 gene can lead to abnormal development of mouse embryonic hearts [11, 12]. The upregulation of CCN1 expression was induced by pressure overload or cardiac ischemia [13–15] as a pathophysiological regulatory factor to mediate myocardial cell apoptosis during myocardial ischemic injury [16]. In acute heart failure, patients with CKD showed an increase in serum CCN1 levels, indicating a worse prognosis [17]. Taken together, these data indicate that CCN1 plays a critical role in the heart. However, the role and potential mechanism of CCN1 in cardiac dysfunction and cardiac remodeling in CKD are unclear.

To explore the role of CCN1 in the cardiac remodeling process caused by CKD, we hope to knock out the CCN1 gene selectively in mouse heart tissue as CCN1-deficient mice suffer embryonic death, partly due to placental vascular insufficiency and impaired integrity [12, 18]. Creatine kinase MM (CKmm) accounts for 70%–85% of creatine phosphokinase activity in the mouse heart tissue [19], effectively increasing the CCN1 knockout efficiency.

CKmm-Cre^+/−^CCN1^flox/flox^ mice were used as experimental animals to establish a 5/6 nephrectomy (5/6 Nx) mouse model, and the structure and function of the mouse heart were analysed 18 months after modeling. The aim of this study was to investigate the role of CCN1 in cardiac remodeling and its potential mechanism in CKD.

## 2. Material and Methods

### 2.1. Animal Experiments

Male CKmm-Cre^−/−^CCN1flox/flox (wild type (WT)) and CKmm-Cre^+/−^CCN1flox/flox (CCN^−/−^) mice were obtained from the animal center of Wenzhou Medical University. All animal experiments were approved by Wenzhou Medical University's Institutional Animal Care and Use Committee. All procedures were performed according to the requirements of the guidelines for the ethical review of laboratory animal welfare of People's Republic of China National Standard. The experimental mice were 14–16 weeks old. The surgical method was followed by Wang et al. [18]. The mice had free access to food and water. They were housed at a constant temperature (22°C–26°C) with a cycle of 12-h light and 12-h dark. All mice were housed in specific pathogen-free conditions. They were divided into four groups: the WT group (SHAM group), WT + CKD group, CCN1^−/−^ group, and CCN1^−/−^ + CKD group (*n* = 6 per group).

### 2.2. Collection of Blood Samples

Blood samples were collected from the eyes of mice in ethylenediaminetetraacetic acid (EDTA) tubes (ST1303, Beyotime Biotech Inc, Shanghai, China). Let it sit overnight in the refrigerator at 4°C. Plasma was separated at 3000 rpm (Thermo America) for 15 min and stored at −80°C until analysis. All mice had blood samples taken 18 months after 5/6 Nx or SHAM surgery.

### 2.3. Detection of Serum Creatinine, Urea Nitrogen, and CYR61/CCN1 in Mice

To quantify the concentrations of creatinine, urea nitrogen, and CCN1 in mouse serum, we performed assays according to the manufacturer's instructions (C011-2-1 and C013-2-1 (Nanjing Jiancheng Biological Engineering Research Institute, Nanjing, China) and the Mouse CYR61 ELISA Kit (ab253223, Abcam, Cambridge, United Kingdom)).

### 2.4. Cardiac Function

We measured cardiac systolic function using two-dimensional echocardiography (Vevo3100, FUJIFILM VisualSonics, Canada) 3 days before killing the mice. After anesthesia, the hair in the precardiac area was removed, and then, the ultrasonic probe was used for ultrasonic detection. Images were acquired in the M-mode and short axis, and related data were recorded. The ejection fraction (EF) was calculated using the following equation: EF = [(LVEDV − LVESV)/LVEDV]∗100%. Moreover, fractional shortening (FS) was calculated using the following equation: FS = [(LVIDd − LVIDs)/LVIDd]∗100%.

### 2.5. Western Blot Analysis

Heart tissue samples (30 mg) and/or cardiomyocyte samples were weighed, ground, and centrifuged at 12,000 rpm for 20 min at 4°C to collect the supernatant. Protein samples were separated by sodium dodecyl sulphate–polyacrylamide gel electrophoresis (SDS–PAGE) gels (Solarbio, Beijing, China) and transferred to nitrocellulose (NC) membranes (Merck, Germany). NC membranes were blocked with 5% nonfat milk solution for 2 h at room temperature, followed by incubation with primary antibodies overnight at 4°C. The membrane was washed three times with Tris-buffered saline (TBS) containing 0.1% Tween-20 (TBST); TBS and Tween-20 were purchased from Solarbio (Solarbio, Beijing, China), and the band was incubated with horseradish peroxidase–conjugated secondary antibody (A0208 and A0216, Beyotime Biotech Inc, Shanghai, China) for 1 h at room temperature. Finally, proteins were detected by enhanced chemiluminescence (Bio-Rad, United States).

### 2.6. Hematoxylin–Eosin (H&E), Masson's Trichrome, Sirius Red, and Rhodamine Staining

Myocardial tissue was fixed in 4% paraformaldehyde (P1110, Solarbio, Beijing, China) for 48 h. After gradient dehydration, the tissues were embedded in paraffin. Subsequently, the tissues were cut into 4-*μ*m sections. Sections were dewaxed and hydrated. The hydrated sections were used for H&E staining (G1120, Solarbio, Beijing, China). Images of each section were taken using a light microscope (200× magnification; Leica, Germany) to evaluate histopathological changes. Paraffin sections (4 *μ*m) were stained with 0.1% Sirius red (G1472, Solarbio, Beijing, China) and Masson trichrome (G1343, Solarbio, Beijing, China) to assess collagen deposition and fibrosis levels, respectively. H9c2 cells were pretreated with si-CCN1 or si-NC (100 nM, RiboBio Company, Guangzhou, China) for 24 h before being incubated with CKD mouse serum. Then, the cells were stained with rhodamine (CA1610, Solarbio, Beijing, China) according to the manufacturer's instructions, which use tetramethylrhodamine isothiocyanate (TRITC)–labeled phalloidin. Phalloidin selectively binds to fibrous actin in animals and plants, making it a powerful tool for studying intracellular actin filaments. Cells were then observed under a Nikon fluorescence microscope (400× magnification; Nikon, Japan).

### 2.7. H9c2 Cell Culture and Treatment

The rat cardiac muscle–derived cell line H9c2 was purchased from Shanghai Institute of Biochemistry and Cell Biology (Shanghai, China). H9c2 cells were maintained in Dulbecco's modified Eagle medium (DMEM) (11965092, Gibco, Shanghai, China) supplemented with 20% fetal bovine serum (FBS) (10099141C, Gibco, Shanghai, China), 100 U/mL penicillin, and 100 mg/mL streptomycin (15140122, Gibco, Shanghai, China) and cultured in a 5% CO_2_ incubator at 37°C. Experiments were performed when the cell density was approximately 70%–80%. We used a filter with a pore size of 0.22 *μ*m to filter impurities from the serum obtained from the WT-CKD group of mice; the obtained mouse serum was mixed with DMEM to make 20% 5/6 Nx mouses' serum; SHAM mouses' serum was obtained in the same way from WT group mice. The experiments were divided into SHAM serum (20% of SHAM mouse serum was added to H9c2 cells for 24 h), CKD serum (20% of 5/6 Nx mouse serum was added to H9c2 cells for 24 h), si-CCN1 + CKD serum (before exposure to 20% of 5/6 Nx mouse serum, si-CCN1 was added to H9c2 cells for 24 h), and si-NC + CKD serum (before exposure to 20% of 5/6 Nx mouse serum, si-NC was added to H9c2 cells for 24 h). Next, Jun amino-terminal kinase (JNK)–specific inhibitor (SP60012), a P38-specific inhibitor (SB203580), and extracellular signal–related kinase (ERK)–specific inhibitor (PD98059) (Sigma, United States) or MAPK agonist (100 nM phorbol 12-myristate 13-acetate (PMA) (Sigma-Aldrich; Merck KGaA)) were dissolved in DMSO and added to the cells for 2 h before exposure to culture medium containing 20% 5/6 Nx mouse serum for 24 h. The final concentration of the three inhibitors used for experiments was 10 *μ*M.

### 2.8. Reagents

DMEM, FBS, and penicillin/streptomycin (pen/strep, 10,000 U/mL and 10,000 *μ*g/mL, respectively) were purchased from the Gibco Company (Shanghai, China). si-CCN1 and si-NC were purchased from the RiboBio Company (Guangzhou, China). A P38-specific inhibitor (SP60012), JNK-specific inhibitor (SB203580), and ERK-specific inhibitor (PD98059) were purchased from Sigma (United States). PMA was purchased from Sigma-Aldrich (Merck KGaA, China). Anti-cyr61/CCN1 antibody (1 *μ*g/mL, NB 100–356) was obtained from Novus Biologicals (Littleton, Colorado, United States). Anti–atrial natriuretic peptide (ANP) (1 *μ*g/mL, sc-515701) and anti–collagen 1 (COL-1) (0.5 *μ*g/mL, sc-293182) were purchased from Santa Cruz Biotechnology (Shanghai, China), and anti–heavy-chain cardiac myosin (MyHC) (1 *μ*g/mL, ab50967) was purchased from Abcam (Cambridge, United Kingdom). In addition, anti–transforming growth factor-*β* (TGF-*β*) (1 *μ*g/*μ*L, ER31210, Huabio, China), anti-GAPDH (34 ng/mL, Cat. No. 5174), and anti-P38 (55 ng/mL, Cat. No. 8690S), anti–phosphorylated (p)-P38 (33 ng/mL, Cat. No. 4511T), anti-JNK (41 ng/mL, Cat. No. 9252S), anti-p-JNK (293 ng/mL, Cat. No. 4668S), anti-ERK (84 ng/mL, Cat. No. 4695S), and anti-p-ERK (502 ng/mL, Cat. No. 4370S) were purchased from Cell Signaling Technology (Danvers, Massachusetts, United States). Goat anti-rabbit secondary antibodies (1 *μ*g/*μ*L, A0208) and goat anti-mouse secondary antibodies (1 *μ*g/*μ*L, A0216) used in the Western blot were obtained from Beyotime (Shanghai, China). The H&E staining kit, Masson's trichrome stain kit, Sirius red kit, and rhodamine staining kit were obtained from Solarbio (Beijing, China). The creatinine ELISA kit (C011-2-1) and urea nitrogen ELISA kit (C013-2-1) were obtained from Nanjing Jiancheng Biological Engineering Research Institute (Nanjing, China). CCN1 concentrations assays (ab253223) were obtained from Abcam (Cambridge, United Kingdom).

### 2.9. Statistical Analysis

All statistical analyses were performed using GraphPad Prism 6.0 software (GraphPad, San Diego, California, United States). All data were tested for normality; if normally distributed, data are shown as the mean ± SD, while if not normally distributed, data are presented as media and interquartile. All data were tested for variance chi-square; if variance chi-square was satisfied, unpaired *t*-test was used to compare the two groups; multiple comparisons were performed by two-way ANOVA; if not, the data were statistically analysed using the Kruskal–Wallis test. For all tests, *p* values < 0.05 were considered significant.

## 3. Results

### 3.1. CCN1 Expression Was Elevated in the Serum and Heart of a 5/6 Nx Mouse Model

**Figure 1:**
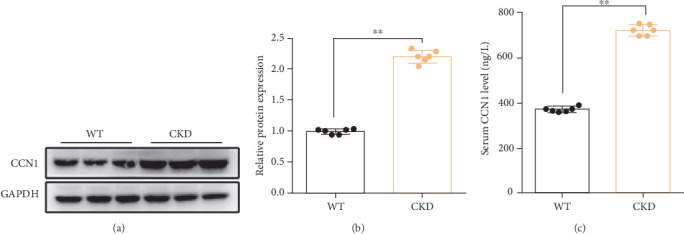
The CCN1 level in CKD mice. (a) The expression level of CCN1 in heart tissue was measured by Western blot analysis. (b) Quantitative analysis of expression levels in heart tissue. (c) The serum CCN1 level in each group. (b, c) Unpaired *t*-test.⁣^∗∗^*p* < 0.01.

In this study, in CKD mice (5/6 Nx mouse model), compared with the WT group, the expression of CCN1 in the serum was increased. We also detected the CCN1 protein level in the heart tissue of mice, and the result was the same as the changes in CCN1 expression in serum (Figures 1(a), 1(b), and 1(c)).

### 3.2. Knockout of CCN1 Can Prevent Myocardial Remodeling in Elderly Mice

**Figure 2:**
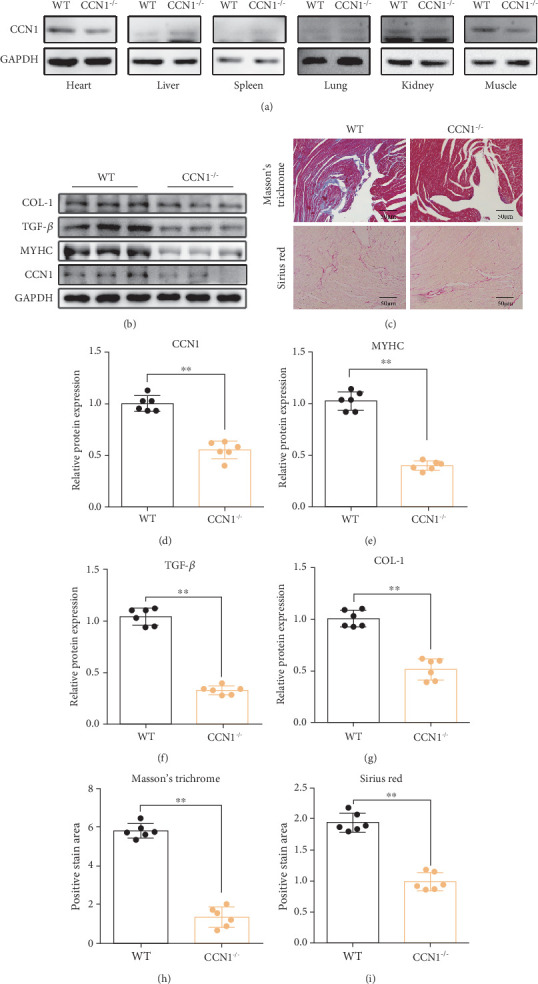
CCN1 deletion prevented cardiac damage. (a) CCN1 expression in different tissues and organs. (b) The expression levels of CCN1, MyHC, TGF-*β*, and COL-1 in heart tissue were measured by Western blot analysis. (c) Myocardial fibrosis was detected by Masson's trichrome staining and Sirius red staining (200× magnification). (d–g) Quantitative analysis of the expression levels of CCN1, MyHC, TGF-*β*, and COL-1 in heart tissue. (h) Quantitative analysis of fibrotic areas (percentage) by Masson's trichrome staining. (i) Quantitative analysis of fibrotic areas (percentage) by Sirius red staining. (d–i) Unpaired *t*-test. ⁣^∗∗^*p* < 0.01.

Previous studies have shown that CCN1 is expressed at high levels in chronic heart failure and myocardial remodeling, and the expression of CCN1 in muscle tissue increases with age [19]. Here, we observed CCN1 expression in different tissues and organs and found that CCN1 was mainly expressed in the heart, kidney, and muscle (Figure 2(a)). Then, we performed Masson's staining and Sirius red staining and detected related protein expression in the heart tissue of mice over 18 months. Compared with the WT group, the CCN1^−/−^ group showed decreased expression of cardiac fibrosis markers (TGF-*β* and COL-1) and the myocardial hypertrophy marker MyHC and lower collagen and fibrous tissue accumulation (Figures 2(b), 2(c), 2(d), 2(e), 2(f), 2(g), 2(h), and 2(i)), suggesting that specific CCN1 inhibition has a protective effect on the heart in elderly mice.

### 3.3. CCN1 Knockout Suppresses Myocardial Remodeling and Improves Cardiac Function in a 5/6 Nx Mouse Model

**Figure 3:**
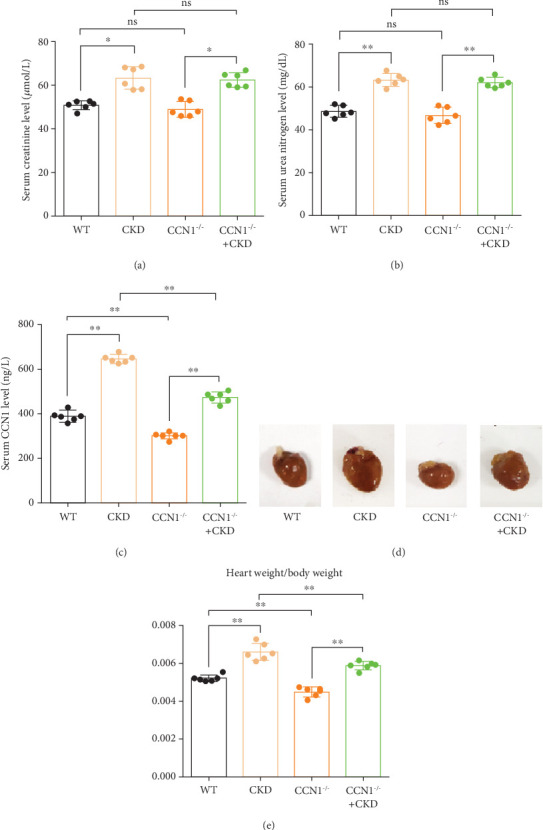
The renal function and appearance of heart in each group. (a) The serum creatinine level in each group. (b) The serum urea nitrogen in each group. (c) The serum CCN1 level in each group. (d) The appearance of heart in mice after 5/6 Nx. (e) Quantitative analysis of heart weight/body weight. (a) Kruskal–Wallis test; (b, c, e) two-way ANOVA. ⁣^∗^*p* < 0.05 and ⁣^∗∗^*p* < 0.01.

**Figure 4:**
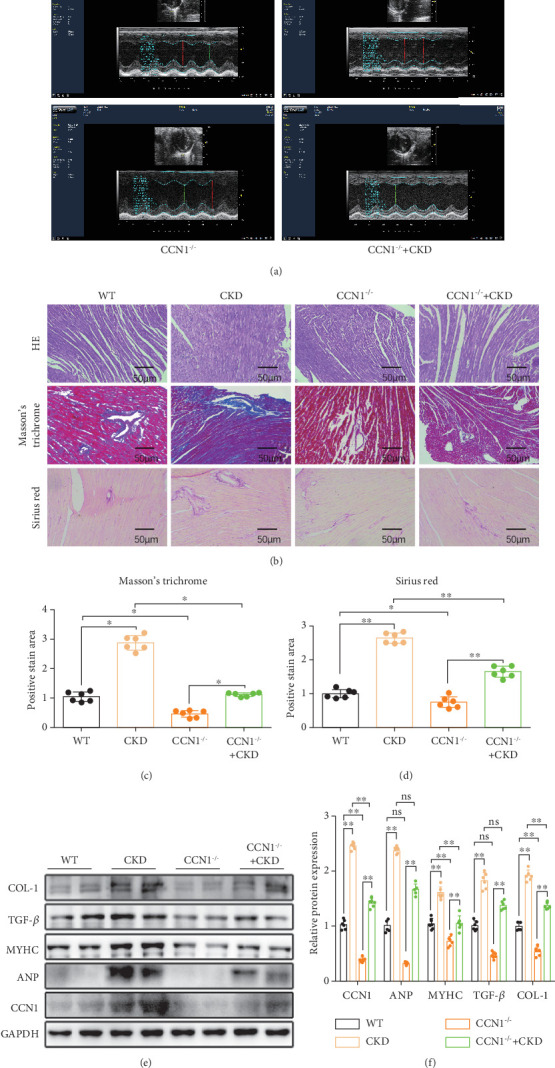
The CCN1 deletion prevented cardiac damage caused by 5/6 Nx. (a) echocardiograms in mice. (b) The myocardial fibrosis was detected by hematoxylin–eosin staining (H&E), Masson's trichrome staining, and Sirius red staining (200× magnification). (c) Quantitative analysis of fibrotic areas (percentage) in Masson's trichrome staining.(d) Quantitative analysis of fibrotic areas (percentage) in Sirius red staining. (e) The expression level of CCN1, ANP, MyHC, TGF-*β*, and COL-1 in heart tissue was measured by Western blot analysis. (f) Quantitative analysis of expression levels in heart tissue. (c) The expression level of ANP and TGF-*β* in (f): Kruskal–Wallis test; (d) the expression level of CCN1, MyHC, and COL-1 in (f) two-way ANOVA. ⁣^∗^*p* < 0.05 and ⁣^∗∗^*p* < 0.01.

After 5/6 Nx for 18 months, the levels of serum creatinine, urea nitrogen, and CCN1 in mice in the WT, WT + CKD, CCN1^−/−^, and CCN1^−/−^ + CKD groups were detected. As a result, compared with the WT or CCN1^−/−^ groups, serum creatinine and serum urea nitrogen were significantly increased in CKD group and the CCN1^−/−^ + CKD group, and compared with the WT or CKD groups, there was no significant difference in CCN1^−/−^ groups or CCN1^−/−^ + CKD group (Figures 3(a) and 3(b)), indicating that the renal function of 5/6 Nx mice was markedly reduced and that specific CCN1^−/−^ had no influence on kidney function. In addition, serum CCN1 levels were detected and significantly increased in the 5/6 Nx model, while decreased in the CCN1^−/−^ or CCN1^−/−^ + CKD group (Figure 3(c)). The heart weight (HW) and body weight (BW) of the mice were measured. We observed that compared with that in the WT group, the HW/BW ratio was significantly increased in the CKD group, while it was effectively reduced in CCN1^−/−^ + CKD mice (Figures 3(d) and 3(e)). Then, we examined the cardiac function of all experimental mice by noninvasive transthoracic echocardiography 3 days before sacrifice. As depicted in Figure 4(a) and Table 1, there was no difference in the heart rate of mice in each group, and there was an obvious reduction in cardiac systolic function as reflected by EF% and FS% after 5/6 Nx in mice, while it was improved in the CCN1^−/−^ or CCN1^−/−^ + CKD group.

As we know, cardiac hypertrophy and fibrosis can result in heart dysfunction; to explore the potential role of CCN1 in cardiac remodeling in mice undergoing 5/6 Nx, we performed H&E, Masson's staining, and Sirius red staining for cardiac histological analysis. After 5/6 Nx, the myocardial structure of mice was disordered, and cardiac fibrosis was obvious compared to that in WT mice; however, these aberrant changes in cardiac tissues were abolished in CCN1^−/−^ mice or CCN1^−/−^ + CKD mice (Figures 4(b), 4(c), and 4(d)). In addition, we also examined relative profibrotic markers (TGF-*β* and COL-1) and cardiac hypertrophy markers (MyHC and ANP) (Figures 4(e) and 4(f)). As expected, the expression of COL-1 and MyHC was markedly increased by CKD and retarded in CCN1^−/−^ + CKD mice; consistent with this, similar results were observed between the WT group and the CCN1^−/−^ group; but about the expression of ANP and TGF-*β*, we found that its expression was increased in the CKD or CCN1^−/−^ + CKD group compared with the WT group or the CCN1^−/−^ group, but there was no significant difference in the CCN1^−/−^ group and WT group or CKD group and CCN1^−/−^ + CKD group. These results could be due to the fact that the datasets for ANP and TGF-*β* did not pass the variance chi-square test, and we used the Kruskal–Wallis test, which is known to be less efficacious than parametric tests.

Overall, in aged CKD mice, CCN1 knockout effectively ameliorated cardiac remodeling.

### 3.4. CCN1 Inhibition Alleviates Cellular Hypertrophy and Fibrosis Induced by CKD Serum in H9c2 Cells

**Figure 5:**
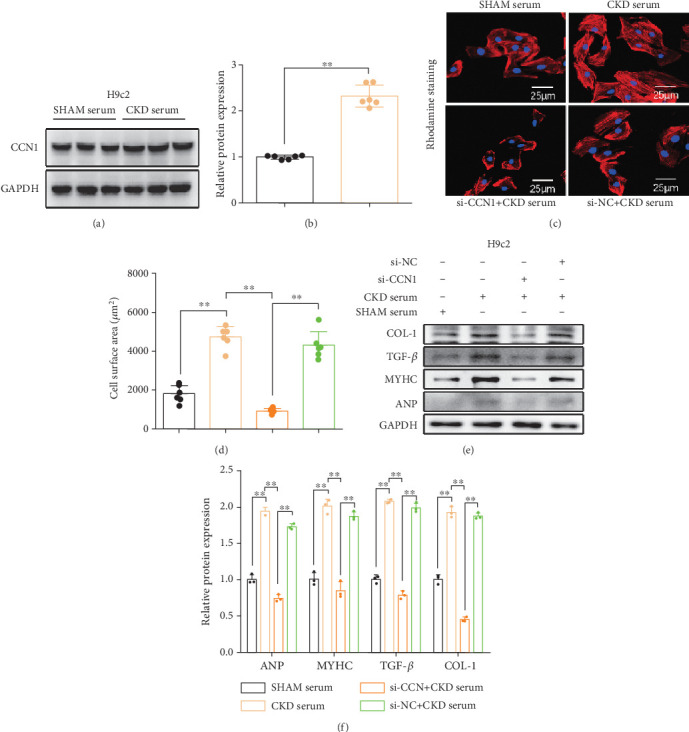
CCN1 inhibition alleviated CKD serum–induced cardiac damage in H9c2 cells. (a) The expression level of CCN1 in heart tissue was measured by Western blot analysis. (b) Quantitative analysis of expression levels in heart tissue. (c) Rhodamine staining in CKD serum–induced H9c2 cells (400× magnification). (d) The quantification of cell size in panel (c). (e) The expression levels of ANP, MyHC, TGF-*β*, and COL-1 in H9c2 cells were measured by Western blot analysis. (f) Quantitative analysis of expression levels in H9c2 cells. (b) Welch's unpaired *t*-test; (d, f) two-way ANOVA. ⁣^∗∗^*p* < 0.01.

Given that CCN1 deficiency in CKD mice improves cardiac systolic function, we speculated that there may be remote crosslinking of the heart and kidney. That is, in CKD, some factors may be secreted into the blood and subsequently act on the heart, eventually leading to cardiac remodeling. Here, we utilized the cell culture system and assessed whether CCN1 mediates CKD-induced cellular hypertrophy and fibrosis. We incubated H9c2 cells with 20% serum from CKD mice for 24 h and found that CCN1 expression was markedly increased in H9c2 cells compared to cells without CKD serum stimulation (Figures 5(a) and 5(b)). Next, to further validate the role of CCN1 in myocardial cells, we transfected siRNA-CCN1 into H9c2 cells, followed by incubation with 20% CKD serum for 24 h. As shown in Figures 5(c) and 5(d), rhodamine staining intuitively showed that si-CCN1 reversed CKD serum–induced cardiomyopathy hypertrophy. Additionally, we detected relative hypertrophy and fibrosis markers, and the results showed that the expression levels of TGF-*β*, COL-1, MyHC, and ANP were significantly decreased in si-CCN1 + CKD serum compared to cells with CKD serum (Figures 5(e) and 5(f)). This suggests that inhibition of CCN1 can alleviate the hypertrophy and fibrosis in H9c2 cells resulting from incubation with CKD serum.

### 3.5. CCN1 Deficiency Suppresses Activation of the MAPK Signaling Pathway In Vivo and In Vitro

**Figure 6:**
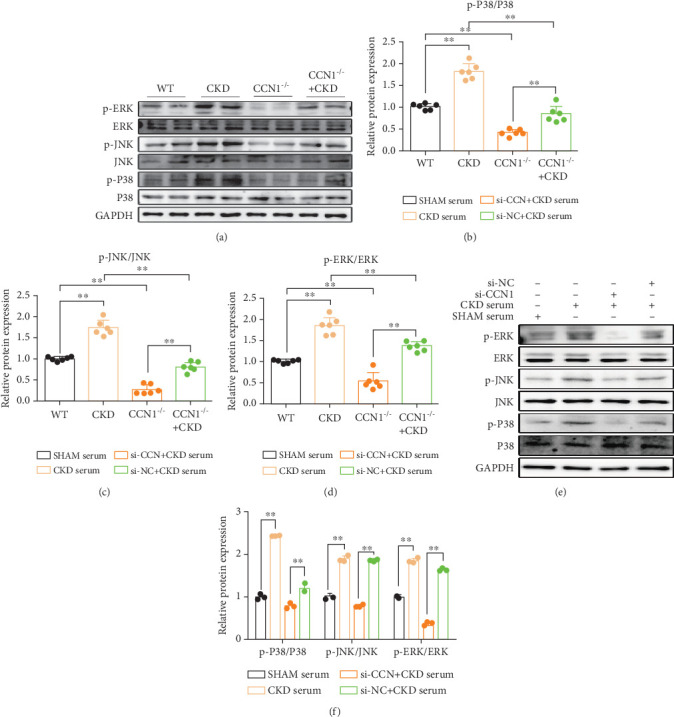
Effects of CCN1 on the MAPK signaling pathway in mice and in CKD serum–induced H9c2 cells. (a) The expression levels of p-P38/P38, p-JNK/JNK, and p-ERK/ERK in heart tissue were measured by Western blot analysis. (b–d) Quantitative analysis of expression levels in heart tissue. (e) The expression levels of p-P38/P38, p-JNK/JNK, and p-ERK/ERK in H9c2 cells were measured by Western blot analysis. (f) Quantitative analysis of expression levels in H9c2 cells. (b–d, f) Two-way ANOVA. ⁣^∗∗^*p* < 0.01.

**Figure 7:**
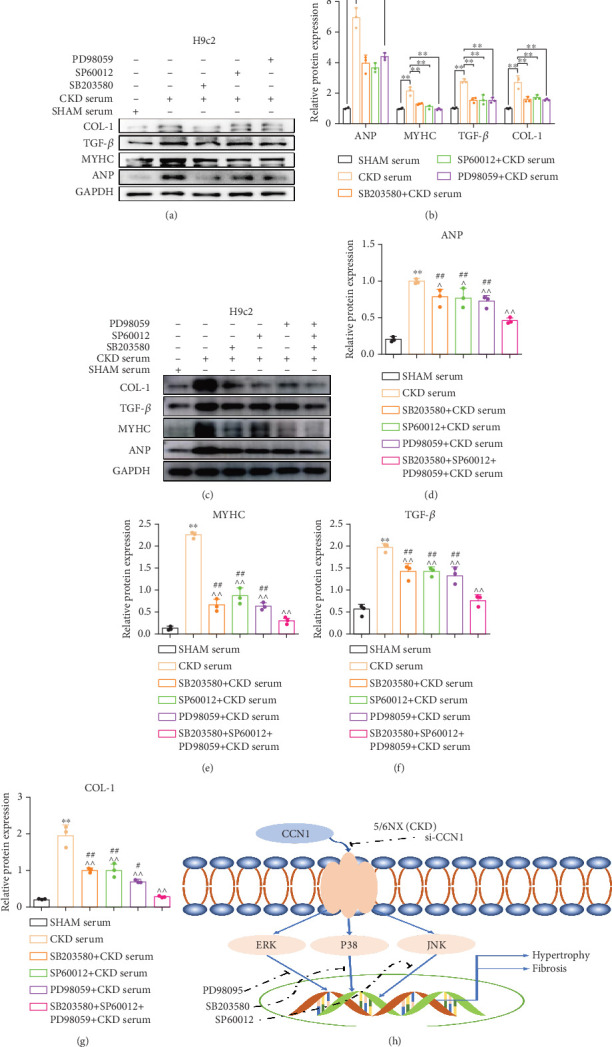
MAPK signaling pathway inhibition alleviated CKD serum–induced cardiac damage. (a) The expression levels of ANP, MyHC, TGF-*β*, and COL-1 in H9c2 cells were measured by Western blot analysis. (b) Quantitative analysis of expression levels in H9c2 cells. (c) The expression levels of ANP, MyHC, TGF-*β*, and COL-1 in H9c2 cells were measured by Western blot analysis. (d–g) Quantitative analysis of expression levels in H9c2 cells. (h) Schematic representation of CCN1 in regulating cardiac remodeling in 5/6 nephrectomized mice. (b) Two-way ANOVA. ⁣^∗∗^*p* < 0.01. (d–g) Two-way ANOVA. ⁣^∗∗^*p* < 0.01 versus the SHAM serum group; ^^^^*p* < 0.01 versus the CKD serum group; ^#^*p* < 0.05, ^##^*p* < 0.01 versus the SB203580 + SP60012 + PD98059 + CKD serum group.

The MAPK pathway plays an important role in cardiac fibrosis and hypertrophy; however, its potential effect on CKD-induced cardiac remodeling needs to be explored. Here, we observed that the MAPK pathway was obviously activated in the heart tissues of mice after 5/6 Nx, as presented by an increase in phos-ERK/ERK, phos-P38/P38, and phos-JNK/JNK, which was blunted by specific CCN1 knockout mice or silencing CCN1 in H9c2 cells. These results showed that CCN1 inhibition also significantly reduced the expression of p-ERK/ERK, p-P38/P38, and p-JNK/JNK in cardiomyocytes caused by CKD (Figures 6(a), 6(b), 6(c), 6(d), 6(e), and 6(f)). In addition, we next determined whether the activation of MAPK was involved in myocardial remodeling in CKD. Then, we pretreated H9c2 cells with relevant inhibitors, including a P38-specific inhibitor (SP60012), a JNK-specific inhibitor (SB203580), and an ERK-specific inhibitor (PD98059) for 2 h and then exposed them to medium containing 20% CKD serum for 24 h. As shown in Figures 7(a) and 7(b), SP60012, SB203580, or PD98059 significantly inhibited the expression of TGF-*β*, COL-1, MyHC, and ANP, respectively, which was stimulated by CKD serum. Moreover, in the group cotreated with the three inhibitors, compared to the group treated with anyone, the expression of these indicators was lower, suggesting a synergistic effect between these pathways (Figures 7(c), 7(d), 7(e), 7(g), and 7(g)). Based on these results, we suggest that in CKD mice, the role of CCN1 in regulating myocardial remodeling may by promoting the activation of the MAPK signaling pathway (Figure 7(h)).

### 3.6. In CKD Serum–Induced H9c2 Cells, MAPK Agonist Partially Reversed the Cardioprotective Effect of CCN Inhibition

**Figure 8:**
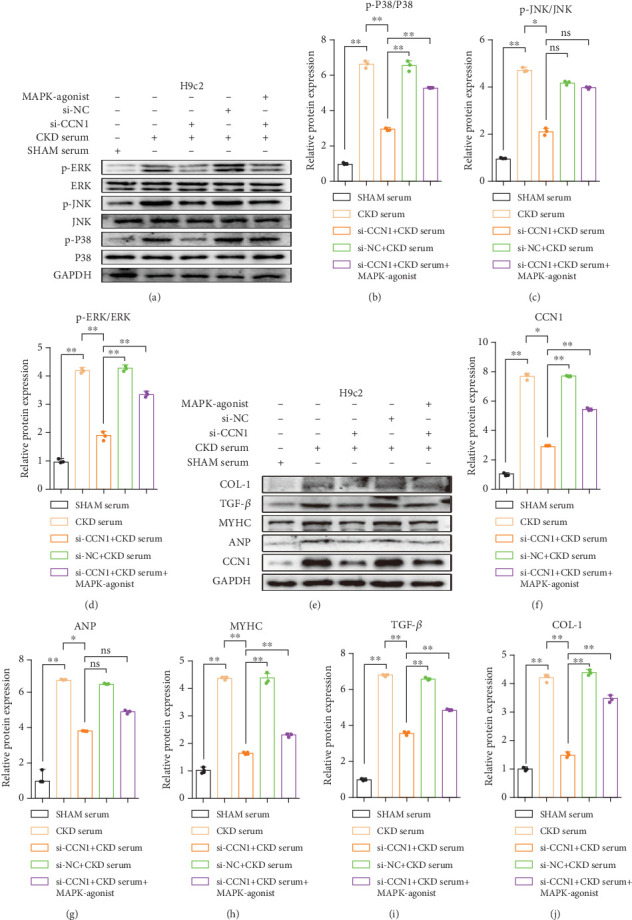
In CKD serum–induced H9c2 cells, MAPK-agonist partially reversed the cardioprotective effect of CCN inhibition. (a) The expression levels of p-P38/P38, p-JNK/JNK, and p-ERK/ERK in H9c2 cells were measured by Western blot analysis. (b–d) Quantitative analysis of expression levels in H9c2 cells. (e) The expression levels of CCN1, ANP, MyHC, TGF-*β*, and COL-1 in H9c2 cells were measured by Western blot analysis. (f–j) Quantitative analysis of expression levels in H9c2 cells. (b, d, f, h, j) Two-way ANOVA; (c, g, i) Kruskal–Wallis test; ⁣^∗^*p* < 0.05 and ⁣^∗∗^*p* < 0.01.

In order to further validate the role of MAPK signal in the si-CCN1-treated and CKD serum–induced H9c2 cells, cells were incubated with the MAPK agonist after being treated with si-CCN1. Fortunately, we found that the expression level of phos-ERK/ERK and phos-P38/P38 was significantly upregulated due to the MAPK-agonist treatment, and the level of phos-JNK/JNK showed no significant difference (Figures 8(a), 8(b), 8(c), and 8(d)). Besides, in MAPK-agonist treatment group, the expression levels of MyHC, TGF-*β*, COL-1 were significantly increased compared with the si-CCN + CKD serum group, while a similar trend was not seen in the expression of ANP (Figures 8(e), 8(f), 8(g), 8(h), 8(i), and 8(j)). For these results for ANP and phos-JNK/JNK, we thought that it was also due to the use of nonparametric tests that do not satisfy the test of chi-squaredness. Taken together, these results indicate that in CKD, CCN1 inhibition regulates cardiac remodeling by downregulating MAPK activation.

## 4. Discussion

In this study, we revealed the role of CCN1 in myocardial remodeling induced by CKD. In mice with CKD, the level of CCN1 is elevated in serum and heart tissues, followed by significant myocardial remodeling, which presents with cardiac fibrosis and hypertrophy and a decrease in cardiac function. By constructing conditional CCN1 knockout mice, we found that CCN1^−/−^ + CKD mice exhibited less cardiac remodeling and better cardiac function than WT + CKD mice. Mechanistically, inactivation of the MAPK signaling pathway was observed under specific CCN1 deficiency or inhibition in both heart tissues and H9c2 cells cultured with serum from CKD mice, while MAPK-related agonist could partially reverse the cardioprotective effect caused by CCN1 inhibition. Moreover, MAPK-related inhibitors markedly abolished the fibrosis and hypertrophy of myocytes induced by serum from CKD mice. Taken together, these results indicate that specific CCN1 knockout or inhibition effectively alleviates myocardial remodeling in CKD mice by suppressing the activation of MAPK signaling, ultimately blocking the progression of cardiorenal syndrome.

CKD is often accompanied by heart failure, especially in patients with end-stage renal disease. The inability of the diseased kidneys to excrete salt and water, as well as abnormal renin secretion, can increase heart load and promote heart failure; in turn, poor renal perfusion due to lower cardiac output aggravates renal failure [20], which means renal–heart crosslinking. Renin–angiotensin system (RAS) activation and increased angiotensin II (Ang II) expression can lead to left ventricular abnormalities, such as cardiac hypertrophy and fibrosis and expansion of cardiomyocyte extracellular matrix, contributing to cardiac dysfunction in CKD [21–24]. Notably, overexpression of Ang II in rat fibroblasts resulted in a significant increase in CCN1 expression [25], indicating that CCN1 may regulate cardiac remodeling. Consistent with this, emerging reports have shown that CCN1 is critical for cardiovascular development during embryonic life and is associated with inflammation, wound healing, damage repair, fibrosis, and cancer in adulthood [26]. Additionally, an increase in CCN1 expression is observed in chronic heart failure and myocardial remodeling tissues [13, 14]. This evidence supports the opinion that CCN1 plays an important role in the structural changes of the heart. However, it remains elusive whether CCN1 participates in the crosstalk between the kidney and heart and in the response to cardiac remodeling. Here, we found an increase in CCN1 expression in the serum and cardiac tissues of mice with CKD. We also observed a significant decrease in cardiac function in mice with CKD, accompanied by enhancement of cardiac hypertrophy and fibrosis. Interestingly, specific knockdown of CCN1 reversed the decline in cardiac function and reduced myocardial fibrosis and hypertrophy in mice with CKD, and the same tendency existed in H9c2 cells treated with serum from mice with CKD. Taken together, these data indicate that cardiac CCN1 expression is obviously augmented and that CCN1 deficiency or suppression can alleviate cardiac remodeling in mice with CKD.

Notably, the MAPK signaling pathway includes ERK1/2, JNK1/2, P38-MAPK, and ERK5, which participate in different cell cycles, such as cell differentiation, proliferation, migration, and apoptosis [27]. Additionally, mounting evidence indicates that the MAPK activation response regulates cardiac hypertrophy and remodeling [28]. For instance, ERK1/2 signaling regulates the balance between eccentric and centripetal cardiac growth [29–31]. P38 activation plays a negative inotropic and restrictive diastolic role in ventricular myocytes [32], and myocardial JNK activation, which directly regulates nuclear factor of activated T cells, is primarily dedicated to the regulation of differentiated heart growth [33]. These data provide support that MAPKs play a critical role in regulating cardiac remodeling under conditions of overload or pathological insults. In addition, MAPKs may be one of the downstream pathways of CCN1 and are activated in HEK293T cells or human epidermal melanocytes [34, 35]. Here, in the hearts of mice with CKD or H9c2 cells induced by serum from CKD, we revealed that MAPKs, including the ERK1/2, JNK1/2, and P38 pathways, were significantly activated, showing the same increasing trend as cardiac CCN1, and were suppressed by the deficiency or inhibition of CCN1. Subsequently, the inhibition of P38, JNK, or ERK1/2 using a relative inhibitor effectively abrogated myocardial hypertrophy and fibrosis in H9c2 cells underlying serum from CKD mice, and based on our experimental results, there may be synergies between them. To further demonstrate the impact of CCN1 on the MAPK pathway. We reduced the expression of CCN1 and conducted experiments using the activation of the MAPK signaling pathway, and the results showed that this agonist can reverse the effect of si-CCN1. These results suggest that MAPK activation, as one of the downstream pathways of CCN1, is involved in the process of cardiac remodeling induced by CKD.

## 5. Conclusion

In summary, our study shows that CCN1 deficiency protects against heart injury induced by CKD in vivo and in vitro. The MAPK pathway exerts a crucial effect on the positive regulatory role of CCN1 in the development of cardiac damage due to CKD. To the best of our knowledge, our results clarify for the first time that (1) CCN1 inhibition possesses an anti–cardiac remodeling role in CKD and (2) provide data to support the underlying mechanism of the crosstalk between the kidney and heart. These findings proved that CCN1 could be a potential therapeutic candidate for retarding cardiac remodeling in CKD and highlight new strategies for the treatment of heart failure.

## Figures and Tables

**Table 1:** Echocardiographic parameters of the CCN1 gene knockout experimental mice.

**Group**	**HR (bpm, ** **n** = 6**)**	**CO (mL/min, ** **n** = 6**)**	**EF (%, ** **n** = 6**)**	**FS (%, ** **n** = 6**)**
WT	463.5 ± 22.8	17.1 ± 2.3	68.0 ± 2.4	37.2 ± 1.8
CKD	432.3 ± 67.0	15.8 ± 1.7	43.6 ± 1.9^∗∗^	21.4 ± 1.1^∗∗^
CCN1^−/−^	446.0 ± 23.8	17.2 ± 4.2	68.1 ± 2.5	37.3 ± 1.7
CCN1^−/−^ + CKD	449.2 ± 33.8	15.4 ± 0.8	52.4 ± 1.0^##$$^	26.5 ± 0.7^##$$^

*Note:* Data presented are as mean ± SD. Two-way ANOVA was used to statistically analyse the data.

Abbreviations: CO: cardiac output, EF: ejection fraction, FS: fractional shortening, HR: heart rate.

⁣^∗∗^*p* < 0.01 versus the WT group.

^##^ *p* < 0.01 versus the CKD group.

^$$^ *p* < 0.01 versus CCN1^−/−^ group.

## Data Availability

The datasets used and/or analysed during the current study are available from the corresponding authors on reasonable request.

## Nomenclature

CKD: chronic kidney disease
CCN1: cysteine-rich protein 61
5/6 Nx: 5/6 nephrectomy
MAPK: mitogen-activated protein kinase
CKmm: creatine kinase MM
EDTA: ethylenediaminetetraacetic acid
DMEM: Dulbecco's modified Eagle medium
FBS: bovine serum
JNK: Jun amino-terminal kinase
ERK: extracellular signal–related kinase
EF: ejection fraction
FS: fractional shortening
NC: nitrocellulose
PMA: phorbol 12-myristate 13-acetate
ANP: atrial natriuretic peptide
COL-1: collagen 1
TGF-*β*: transforming growth factor-*β*
MyHC: heavy-chain cardiac myosin
HW: heart weight
BW: body weight
SDS–PAGE: sodium dodecyl sulphate–polyacrylamide gel electrophoresis
TBS: Tris-buffered saline
RAS: renin–angiotensin system
Ang II: angiotensin II

## References

[1] Levin A., Tonelli M., Bonventre J. (2017). Global kidney health 2017 and beyond: a roadmap for closing gaps in care, research, and policy. *The Lancet*.

[2] Lekawanvijit S., Krum H. (2014). Cardiorenal syndrome: acute kidney injury secondary to cardiovascular disease and role of protein-bound uraemic toxins. *The Journal of Physiology*.

[3] Boor P., Floege J. (2011). Chronic kidney disease growth factors in renal fibrosis. *Clinical and Experimental Pharmacology & Physiology*.

[4] Zanoli L., Lentini P., Briet M. (2019). Arterial stiffness in the heart disease of CKD. *Journal of the American Society of Nephrology*.

[5] Vanholder R., Fouque D., Glorieux G. (2016). Clinical management of the uraemic syndrome in chronic kidney disease. *The Lancet Diabetes and Endocrinology*.

[6] Arem R. (1989). Hypoglycemia associated with renal failure. *Endocrinology and Metabolism Clinics of North America*.

[7] Cohen G. (2020). Immune dysfunction in uremia 2020. *Toxins*.

[8] Grzeszkiewicz T. M., Lindner V., Chen N., Lam S. C. T., Lau L. F. (2002). The angiogenic factor cysteine-rich 61 (CYR61, CCN1) supports vascular smooth muscle cell adhesion and stimulates chemotaxis through integrin *α*_6_*β*_1_ and cell surface heparan sulfate proteoglycans. *Endocrinology*.

[9] Franzen C. A., Chen C. C., Todorović V., Juric V., Monzon R. I., Lau L. F. (2009). Matrix protein CCN1 is critical for prostate carcinoma cell proliferation and TRAIL-induced apoptosis. *Molecular Cancer Research*.

[10] Lau L. F. (2011). CCN1/CYR61: the very model of a modern matricellular protein. *Cellular and Molecular Life Sciences*.

[11] Mo F. E., Lau L. F. (2006). The matricellular protein CCN1 is essential for cardiac development. *Circulation Research*.

[12] Mo F. E., Muntean A. G., Chen C. C., Stolz D. B., Watkins S. C., Lau L. F. (2002). CYR61 (CCN1) is essential for placental development and vascular integrity. *Molecular and Cellular Biology*.

[13] Hilfiker-Kleiner D., Kaminski K., Kaminska A. (2004). Regulation of proangiogenic factor CCN1 in cardiac muscle: impact of ischemia, pressure overload, and neurohumoral activation. *Circulation*.

[14] Zhao J., Zhang C., Liu J. (2018). Prognostic significance of serum cysteine-rich protein 61 in patients with acute heart failure. *Cellular Physiology and Biochemistry*.

[15] Bonda T. A., Kamiński K. A., Dziemidowicz M. (2012). Atrial expression of the CCN1 and CCN2 proteins in chronic heart failure. *Folia Histochemica et Cytobiologica*.

[16] Hsu P. L., Su B. C., Kuok Q. Y., Mo F. E. (2013). Extracellular matrix protein CCN1 regulates cardiomyocyte apoptosis in mice with stress-induced cardiac injury. *Cardiovascular Research*.

[17] Liu C., Liang W., He X. (2020). Prognostic value of cysteine-rich protein 61 combined with n-terminal pro-B-type natriuretic peptide for mortality in acute heart failure patients with and without chronic kidney disease. *Cardiorenal Medicine*.

[18] Wang X., Chaudhry M. A., Nie Y., Xie Z., Shapiro J. I., Liu J. (2017). A mouse 5/6^th^ nephrectomy model that induces experimental uremic cardiomyopathy. *Journal of Visualized Experiments*.

[19] Du J., Klein J. D., Hassounah F., Zhang J., Zhang C., Wang X. H. (2014). Aging increases CCN1 expression leading to muscle senescence. *American Journal of Physiology. Cell Physiology*.

[20] Banerjee D., Rosano G., Herzog C. A. (2021). Management of heart failure patient with CKD. *Clinical Journal of the American Society of Nephrology*.

[21] Travers J. G., Kamal F. A., Robbins J., Yutzey K. E., Blaxall B. C. (2016). Cardiac fibrosis: the fibroblast awakens. *Circulation Research*.

[22] Takawale A., Sakamuri S. S., Kassiri Z. (2015). Extracellular matrix communication and turnover in cardiac physiology and pathology. *Comprehensive Physiology*.

[23] Espira L., Czubryt M. P. (2009). Emerging concepts in cardiac matrix biology. *Canadian Journal of Physiology and Pharmacology*.

[24] Raizada V., Hillerson D., Amaram J. S., Skipper B. (2012). Angiotensin II-mediated left ventricular abnormalities in chronic kidney disease. *Journal of Investigative Medicine*.

[25] Liu B., Yu J., Taylor L., Zhou X., Polgar P. (2006). Microarray and phosphokinase screenings leading to studies on ERK and JNK regulation of connective tissue growth factor expression by angiotensin II 1a and bradykinin B2 receptors in Rat1 fibroblasts. *Journal of Cellular Biochemistry*.

[26] Kim K. H., Won J. H., Cheng N., Lau L. F. (2018). The matricellular protein CCN1 in tissue injury repair. *Journal of Cell Communication and Signaling*.

[27] Sun Y., Liu W. Z., Liu T., Feng X., Yang N., Zhou H. F. (2015). Signaling pathway of MAPK/ERK in cell proliferation, differentiation, migration, senescence and apoptosis. *Journal of Receptor and Signal Transduction Research*.

[28] Rose B. A., Force T., Wang Y. (2010). Mitogen-activated protein kinase signaling in the heart: angels versus demons in a heart-breaking tale. *Physiological Reviews*.

[29] Maillet M., van Berlo J. H., Molkentin J. D. (2013). Molecular basis of physiological heart growth: fundamental concepts and new players. *Nature Reviews Molecular Cell Biology*.

[30] Kehat I., Davis J., Tiburcy M. (2011). Extracellular signal-regulated kinases 1 and 2 regulate the balance between eccentric and concentric cardiac growth. *Circulation Research*.

[31] Mutlak M., Kehat I. (2015). Extracellular signal-regulated kinases 1/2 as regulators of cardiac hypertrophy. *Frontiers in Pharmacology*.

[32] Liao P., Georgakopoulos D., Kovacs A. (2001). The *in vivo* role of p38 MAP kinases in cardiac remodeling and restrictive cardiomyopathy. *Proceedings of the National Academy of Sciences of the United States of America*.

[33] Liang Q., Bueno O. F., Wilkins B. J., Kuan C. Y., Xia Y., Molkentin J. D. (2003). c-Jun N-terminal kinases (JNK) antagonize cardiac growth through cross-talk with calcineurin-NFAT signaling. *The EMBO Journal*.

[34] Wang J., Fu D., Senouthai S., Jiang Y., Hu R., You Y. (2019). Identification of the transcriptional networks and the involvement in angiotensin II-induced injury after CRISPR/Cas9-mediated knockdown of Cyr61 in HEK293T cells. *Mediators of Inflammation*.

[35] Xu Z., Chen L., Jiang M., Wang Q., Zhang C., Xiang L. F. (2018). CCN1/Cyr61 stimulates melanogenesis through integrin *α*6*β*1, p38 MAPK, and ERK1/2 signaling pathways in human epidermal melanocytes. *The Journal of Investigative Dermatology*.

